# Physical Exercise Improves Cognitive Function Together with Microglia Phenotype Modulation and Remyelination in Chronic Cerebral Hypoperfusion

**DOI:** 10.3389/fncel.2017.00404

**Published:** 2017-12-22

**Authors:** Ting Jiang, Liying Zhang, Xiaona Pan, Haiqing Zheng, Xi Chen, Lili Li, Jing Luo, Xiquan Hu

**Affiliations:** Department of Rehabilitation Medicine, The Third Affiliated Hospital, Sun Yat-sen University, Guangzhou, China

**Keywords:** physical exercise, vascular cognitive impairment, remyelination, M2 microglia, CX3CL1/CX3CR1 axis

## Abstract

Myelin is closely associated with cognitive function and is extremely vulnerable to damage in ischemic cerebrovascular diseases. The failure of remyelination is mainly due to limitations in oligodendrocyte progenitor cells (OPCs) differentiation in the damaged area. Previous studies have shown that physical exercise can improve vascular cognitive impairment, but whether it can reverse the defect in remyelination during ischemic injury and the underlying mechanism remains unclear. In this study, we observed the effects of physical exercise on chronic cerebral hypoperfusion (CCH) established by bilateral carotid artery occlusion. The cognitive function, myelin integrity, OPCs proliferation and differentiation, as well as microglia polarization were analyzed at 28 days after CCH. Besides, the expression of CX3CL1/CX3CR1 axis and activation of mitogen-activated protein kinase (MAPK) signal cascades were also evaluated. We found that physical exercise improved the cognitive function of rats with CCH, alleviated myelin injury, triggered OPCs proliferation and differentiation, facilitated microglia polarization toward M2, augmented the expression of CX3CL1/CX3CR1 axis, and reduced ERK and JNK phosphorylation. The results indicated that physical exercise improved the cognitive function of rats with CCH, possibly through microglial phenotype modulation and enhancement of oligodendrocytegenesis and remyelination. Moreover, the CX3CL1/CX3CR1 axis played an important role in this process by mediating ERK- and JNK-dependent pathways.

## Introduction

Myelin is an insulating sheath around the axons formed by oligodendrocytes in the central nervous system (CNS) and plays an important role in the development and maintenance of cognitive function. Myelin can promote rapid conduction and synchronization of optimal function-dependent neural networks by increasing the action potential transmission speed, and improve the capabilities of connectivity and information processing in the brain (Haroutunian et al., 2014). However, myelin is vulnerable to damage in various neurological diseases, such as ischemic cerebrovascular diseases (Pantoni et al., 1996; Sun et al., 2013). In the brain, myelin is mainly distributed in white matter (WM), which receives a disproportionately low blood supply and little collateral circulation. Thus, WM is particularly susceptible to ischemic insults, and this type of damage is an important characteristic of vascular cognitive impairment (Miyamoto et al., 2010). Oligodendrocyte progenitor cells (OPCs) remain in the CNS even after adulthood and provide the opportunity for remyelination after injury (Reynolds and Hardy, 1997; Levison et al., 1999). OPCs can respond immediately to myelin damage through proliferation and recruitment to the demyelinated area, and then differentiate into myelinating oligodendrocytes to restore myelin (Gensert and Goldman, 1997; Franklin and Ffrench-Constant, 2008). However, OPCs are often not recruited to the injured area or do not differentiate into myelinating oligodendrocytes, leading to ineffective remyelination during disease progression (Fancy et al., 2009; Sun et al., 2013).

Various human and animal studies have shown that physical exercise can improve cognitive function, but the mechanism remains unclear (Hillman et al., 2008). At this time, few studies have investigated the effect of physical exercise on oligodendrocyte lineage and remyelination under brain ischemia. The positive effects of physical exercise on OPCs proliferation, differentiation and myelination have been supported in previous reports (Tomlinson et al., 2016). For example, physical exercise may promote oligodendrocyte lineage development and myelination by improving neuronal activity and releasing neurotrophic factors, such as brain-derived neurotrophic factor (BDNF) and insulin-like growth factor-1 (IGF1; Jensen and Yong, 2016; Tomlinson et al., 2016). Other studies have shown that voluntary exercise could promote hindbrain myelination and extend the long-term survival of Snf2h-null mice by facilitating vascular growth factor release (Alvarez-Saavedra et al., 2016). However, further studies are required to explore whether physical exercise can reverse the defect in OPCs recruitment and differentiation in the area of myelin damage and resolve the underlying mechanisms for efficient remyelination during pathological progression.

Although there may not be a single mechanism leading to physical exercise-induced changes in the CNS, many studies have suggested that microglia regulation may be a key mechanism of the beneficial effects exerted by physical exercise (Jensen and Yong, 2014). Microglia is a key modulator in regulating the immune response and pathophysiological processes of the CNS and can be activated rapidly during the very early stage in almost all CNS diseases. These activated microglia can be divided into two phenotypes: classic inflammatory activation (M1) or alternative neuroprotective activation phenotype (M2; Franco and Fernández-Suárez, 2015; Nakagawa and Chiba, 2015). Microglia phenotype modulation has been suggested to be the most effective approach to enhance remyelination (Franklin, 2002; Franklin and Ffrench-Constant, 2008). M1 microglia may cause oligodendrocytes apoptosis and inhibit subsequent remyelination by increasing antigen presentation and producing toxic cytokines. In contrast, M2 microglia may have a neuroprotective effect on oligodendrocytegenesis and remyelination by alleviating local inflammation, clearing cellular debris and releasing neurotrophic factors (Miron et al., 2013). Physical exercise has been shown to mitigate microglia chronic activation and shift it toward a neuroprotective phenotype (Kohman et al., 2012). Whether it has positive effects on remyelination under ischemic injury by affecting the activation and phenotype transition of microglia requires further investigation.

The CX3CL1/CX3CR1 axis, an important signaling pathway in regulating microglia activation, plays a role in maintaining microglia in a resting state and suppressing the neurotoxic effect of activated microglia (Cardona et al., 2006; Ransohoff and El Khoury, 2015). In addition, the CX3CL1/CX3CR1 axis can induce microglia polarization to a neuroprotective phenotype (Vukovic et al., 2012). In the CNS, CX3CL1 is mainly expressed in normal neurons, while its receptor CX3CR1 is exclusively expressed by microglia. The expression of CX3CL1/CX3CR1 signaling changes immediately when suffering from damage, resulting in microglia activation and neurotoxicity (Harrison et al., 1998; Rogers et al., 2011). At this time, whether the CX3CL1/CX3CR1 axis is beneficial or destructive during ischemic injury remains unclear (Rogers et al., 2011).

In this study, we explored whether physical exercise can rescue chronic cerebral hypoperfusion (CCH)-induced cognitive impairment by switching microglia to a neuroprotective phenotype and producing a more favorable microenvironment for oligodendrocytegenesis and remyelination. Moreover, we further explored the role of the CX3CL1/CX3CR1 axis and its downstream mitogen-activated protein kinase (MAPK) signaling in the process of regulating microglia activation.

## Materials and Methods

### Animals and Subgrouping

**Figure 1:**
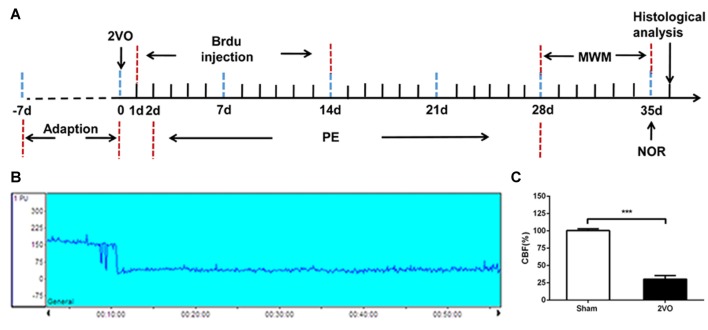
Experimental schedule and CBF decreased after 2VO. **(A)** The experimental schedule to observe the effects of physical exercise on chronic cerebral hypoperfusion (CCH). **(B)** Representative graph of CBF changes before and after 2VO. **(C)** Changes in CBF in sham and 2VO rats. Data represent the means ± standard error of mean (SEM). *n* = 10. ***P < 0.001. 2VO, two-vessel occlusion; PE, physical exercise; MWM, Morris water maze; NOR, novel object recognition; CBF, cerebral blood flow.

A total of 106 male Wistar rats (weight, 280–320 g) were used in this study. Animals were maintained in groups with free access to food and drink. The feeding environment was maintained for 12 h of circadian circulation, and room temperature was constantly maintained at 23 ± 1°C. Animals were adapted to the environment for 1 week before experiments. All animal treatments and experiments were approved by the Institutional Animal Ethical Committee Sun Yat-sen University, and treatment conformed to the Guide for the Care and Use of Laboratory Animals of the National Institute of Health (Publication No. 80-23, revised 1996). Rats were randomly divided into sham group and two-vessel occlusion (2VO) group, then the sham group was further subdivided into sham group (*n* = 28) and sham with physical exercise group (*n* = 22); the successful model of 2VO rats were randomly divided into 2VO control group (*n* = 28) and 2VO with physical exercise group (*n* = 28). The experimental schedule was presented in Figure 1A.

### Two-Vessel Occlusion Surgery and Cerebral Blood Flow Measurement

2VO was performed to establish the CCH model; the cerebral blood flow (CBF) before and after 2VO were measured by laser Doppler flowmetry. After being anesthetized with 2% pentobarbital, rats were fixed on a stereotaxic instrument. Using a scalp incision along the midline, a burr hole was made with an electric drill on the right frontal region (0.8 mm posterior and 3.4 mm lateral to the Bregma) to set a placement device fixed by cyanoacrylate adhesives for the contact probe (Tomimoto et al., 2003). The rat was then turned over and a middle incision was made in the neck. The bilateral common carotid arteries were carefully separated and double ligated with a 4–0 silk-suture, and then cut between the middle of the double ligations. The sham group was subjected to the same procedure but without common carotid arteries ligation (Wakita et al., 1994). During the procedure, the body temperature of the rat was maintained at 37°C. The mean CBF values were expressed as a percentage of the baseline value. The successful 2VO model was defined as a decline in CBF to approximately 30% of the baseline before surgery (Choy et al., 2006).

### Physical Exercise and Bromodeoxyuridine Injections

Rats in the physical exercise group were placed into a programmable, motorized wheel apparatus (diameter, 21 cm; length, 40 cm) and began to perform running exercise 48 h after 2VO. The initial speed was 5 rev/min for 20 min, and the training intensity gradually increased over time. The running speed increased to 7 rev/min on day 7, 10 rev/min on day 14, 15 rev/min on day 21 and 20 rev/min on day 28. The frequency of exercise training is twice a day, 6 days per week. The control group and sham group were housed in a standard cage without any targeted training.

5-bromo-20-deoxyuridine (BrdU) was injected intraperitoneally twice a day for each group until 14 days at 24 h after 2VO.

### Morris Water Maze Test

The Morris water maze (MWM) was used to evaluate spatial learning and memory of each group at 28 days after 2VO. The device consisted of a 150-cm-diameter round pool filled with water to a depth of 35 cm. The water temperature was maintained at 22 ± 2°C. In the target quadrant (QIII) of the pool, a 15-cm-diameter escape platform was placed approximately 2 cm below the water surface. A camera was mounted directly above the center of the maze to record animal behavior, and the information was analyzed using software. The rats firstly received place navigation test for five consecutive days. Rats were gently put into the water and released facing the wall from one of four quadrants in a random order. They were allowed to find the escape platform for 60 s, and the latency to escape onto the platform was recorded. If a rat failed, it would be guided onto the platform by a stick and its latency time was recorded as 60 s. Regardless of being found on the platform or not, each rat stayed on the platform for 10 s. Rats were trained four times a day, with inter-trial intervals of approximately 20 min. The escape latency were measured and analyzed. The day after the place navigation test, a 60-s spatial probe test was conducted with the platform removed. The times of rats crossing the platform area and the dwell time in the target quadrant where the platform was located before were recorded during the training. In addition, the swimming speed was recorded to evaluate motor function of rats for 6 days.

### Novel Object Recognition Test

The novel object recognition (NOR) test is commonly used to evaluate non-spatial memory between the prefrontal and subcortical circuits. The experiment was performed in a 72 × 72 × 35 cm^3^ transparent open-field box. The day before testing, rats were habituated to freely explore the experimental apparatus for 5 min without objects. Rats were then given a session of two trials with an inter-trial interval of approximately 1 h. In the first trial, rats were placed into the experimental apparatus and allowed to explore two identical objects located on the diagonal of the box equidistant and symmetrical for 5 min. During the second trial, rats were put into the box again; however, one of the familiar objects was replaced by a novel object with different colors and shapes; the rats were left in the apparatus for 5 min. The box and objects were carefully cleaned with 70% ethanol after each trial to clear the smell of rats. A camera was installed directly above the experimental apparatus to record the time that animals explored familiar objects (F) and novel objects (N). The discrimination ratio was calculated as N/(N+F) × 100% to compare differences among groups.

### Tissue Preparation for Histochemistry

At 7, 14, or 28 days after 2VO, 6 rats from each group were deeply anesthetized with 2% pentobarbital and perfused transcardially with 0.9% saline at 4°C, followed by 4% paraformaldehyde in phosphate buffer. Subsequently, brains were quickly removed and kept in 4% paraformaldehyde for 24 h at 4°C. For dehydration, brains were incubated in 20% and 30% sucrose successively. Then brains were embedded with OCT and cut into 10-μm-thick slices with a cryostat microtome (CM1900, Leica, Germany) in the coronal plane for luxol fast blue (LFB) staining, immunohistochemical staining and immunofluorescence.

### Luxol Fast Blue Staining

LFB staining was used to assess myelin content after 2VO. Frozen sections were placed in 1:1 alcohol/chloroform overnight and then hydrated back with 95% ethyl alcohol. The slices were placed into LFB solution and incubated at 60°C overnight. Subsequently, slices were rinsed with 95% ethanol and double-distilled water in sequence. In the differentiation step, slices were placed in a lithium carbonate solution and then followed by 70% ethanol and double-distilled water for 10 s. Using a microscope, we explored whether the WM was sharply defined. If necessary, we repeated the above differentiation steps. After differentiation, the slices were rinsed with 100% alcohol for 5 min, xylene for 5 min, mounted with a neutral balsam and finally observed under the microscope camera.

### Immunohistochemical Staining

Iba1 immunohistochemistry was performed to detect the different morphological phenotypes of microglia. Briefly, sections were pretreated with hot (85°C) citrate buffer for 5 min for antigen retrieval followed by blocking buffer consisting of 3% BSA and 0.3% Triton X-100 in PBS for 1 h at room temperature. Next, sections were incubated with rabbit anti-Iba1 (1:500, 019-19741, Wako, Japan) primary antibody overnight at 4°C. The following day, sections were incubated with HRP-labeled goat anti-rabbit secondary antibody (1:500, K5007, Dako, Denmark) for 1 h at room temperature. Subsequently, sections were treated with 3,30-diaminobenzidine (DAB; K3468, Dako, Denmark). After counterstained with Hematoxylin and dehydrated by gradient ethanol elution, sections were coverslipped with neutral balsam and observed under the microscope camera (BX63; Olympus).

### Immunofluorescence Staining

Immunofluorescence staining was performed as follows: for BrdU immunostaining, brain sections were first incubated in 2 N HCl at 37°C for 30 min and neutralized with 0.1 M borate solution for 10 min. For double immunofluorescence staining, all sections were pretreated with hot (85°C) citrate buffer for 5 min for antigen retrieval followed by immunol-staining blocking buffer (P0102, Beyotime, China) for 1 h at room temperature. Next, sections were incubated with mixtures of rabbit anti-myelin basic protein (anti-MBP; 1:500, ab40390, Abcam, UK) and mouse anti-SMI32 (1:500, NE1023, Calbiochem, USA); rat anti-BrdU (1:200, ab6326, Abcam, UK) and rabbit anti-NG2 (1:200, AB5320, Millipore, USA); or mouse anti-APC (1:200, OP80, Calbiochem, USA); rabbit anti-Iba1 (1:500, 019-19741, Wako, Japan), and mouse anti-IGF1 (1:50, 05-172, Millipore, USA); mouse anti-Iba1 (1:100, MABN92, Millipore, USA), and rabbit anti-CD86 (1:200, ab209896, Abcam, UK) or rabbit anti-CX3CR1 (1:500, ab8021, Abcam, UK) primary antibody overnight at 4°C. The following day, sections were incubated with mouse anti-rabbit IgG (1:1000, #4408/4409 Cell Signaling Technology, USA), rabbit anti-mouse IgG (1:1000, #4412/4413, Cell Signaling Technology, USA) and donkey anti-rat IgG (1:1000, A21209, Invitrogen, USA) for 1 h at room temperature. After rinsing, sections were mounted with a DAPI-containing antifade solution. Fluorescence signals were then observed under a microscope (BX63; Olympus) and a confocal microscope (Zeiss; LSM800).

### Quantitative Real-Time Polymerase Chain Reaction (qRT-PCR)

Five rats from each group were randomly selected and anesthetized. Then the brain tissues were removed quickly from the corpus callosum and the mRNA expression level of M1 (*CD86, iNOS*) and M2 (*CD206, Arg1*) microglia markers, inflammatory factors (*IL-1β, TNFα, IL-4, IL-10*) and neurotrophic factors (*IGF1, BDNF*) were measured by real-time polymerase chain reaction (RT-PCR). Briefly, the total RNA was extracted with a Trizol reagent and subjected to complementary DNA synthesis with the TOYOBO cDNA synthesis kit (TOYOBO, Japan) according to the manufacturer’s instructions. The total PCR system contained cDNA, SYBR Green DNA polymerase, RNAse-free water and primers. The experiment was repeated three times and the primer sequences are listed in Table 1.

**Table 1:** Forward and reverse sequences of the used primers.

Gene	Primer sequences (5′-3′)
*CD86*	F:GACACCCACGGGATCAATTA	R:GCCTCCTCTATTTCAGGTTCAC
*iNOS*	F:GATAAAGGGACAGCGTCAGC	R:CCTTCGGGCCAAAGATCCTG
*CD206*	F:ACTGCGTGGTGATGAAAGG	R:TAACCCAGTGGTTGCTCACA
*Arg-1*	F:TGGCGTTGACCTTGTCTTGT	R:TTTGCTGTGATGCCCCAGAT
*IL-1β*	F:GGCAACTGTCCCTGAACT	R:TCCACAGCCACAATGAGT
*TNF-α*	F:GACCCTCACACTCAGATCATCTTCT	R:TGCTACGACGTGGGCTACG
*IL-4*	F:CGTGATGTACCTCCGTGCTT	R:GTGAGTTCAGACCGCTGACA
*IL-10*	F:TTGAACCACCCGGCATCTAC	R:CCAAGGAGTTGCTCCCGTTA
*BDNF*	F:TTGAGCACGTGATCGAAGAGC	R:GTTCGGCATTGCGAGTTCCAG
*IGF-1*	F:GTCGTCTTCACATCTCTTCTACCT	R:CAACACTCATCCACAATGCCCG
*GAPDH*	F:CTGCTCCTCCCTGTTCTA	R:CAATGTCCACTTTGTCAC

### Western Blot Analysis

Western blot was performed as follows: briefly, the remaining five rats from each group were selected for western blot analysis. After deep anesthetized, the brain tissues were removed quickly from the corpus callosum. Total proteins were extracted using tissue protein extraction reagents. Total tissue proteins (20 μg/lane) were loaded and separated on sodium dodecyl sulfate (SDS)-polyacrylamide gels and then transferred onto polyvinylidene fluoride (PVDF) membranes. Subsequently, membranes were incubated overnight with primary antibodies; namely, CX3CL1 (1:500, ab25088, Abcam, UK), CX3CR1 (1:500, ab8021, Abcam, UK), P38 (1:500, #8690, Cell Signaling Technology, USA), P-P38 (1:500, #4511, Cell Signaling Technology, USA), JNK (1:500, #9252, Cell Signaling Technology, USA), P-JNK (1:500, #4668, Cell Signaling Technology, USA), ERK (1:500, #4695, Cell Signaling Technology, USA), P-ERK (1:500, #4370, Cell Signaling Technology, USA) and GAPDH (1:500, BM3876, Boster, China). Next, membranes were incubated with horse-radish peroxidase-labeled goat anti-rabbit and goat anti-mouse secondary antibody (1:5000, BA1054/BA1050, Boster, China) for 1 h. The ECL Western blot detection kit and an enhanced chemiluminescence system was used to examine the protein bands. The experiment was repeated three times and all measured protein levels were quantified by densitometry.

### Statistical Analysis

SPSS20.0 was used for statistical analysis. The data were expressed as the mean ± standard error of mean (SEM). The mortality rates were examined by fisher’s exact test. MWM results were analyzed by repeated measures one-way analysis of variance (ANOVA). The results of LFB staining, immunofluorescence, RT-PCR and Western blotting were evaluated by ANOVA. A *p*-value < 0.05 was considered statistically significant.

## Results

### Cerebral Blood Flow Decreased after 2VO

The regional CBF was monitored by laser Doppler flowmetry before and after 2VO. Based on our results, a decrease in CBF was not obvious when only one side of the common carotid artery was ligated, and soon returned to the baseline level. However, the blood flow decreased immediately from the baseline level of 185.91 ± 16.14 PU to 55.39 ± 13.92 PU when both sides were ligated (Figure 1B). The CBF decreased to about 30% of the baseline level before 2VO.There was no significant change in CBF in the sham rats (Figure 1C). Then the sham group was further subdivided into sham group and sham + PE group, the successful model of 2VO rats were randomly divided into 2VO control group and 2VO + PE group, and there were no significant differences in mortality rates between the two groups of sham and sham + PE group, and between 2VO and 2VO + PE group (Supplementary Table S1).

### Physical Exercise Improved the Cognitive Function of Rats with Chronic Cerebral Hypoperfusion

**Figure 2:**
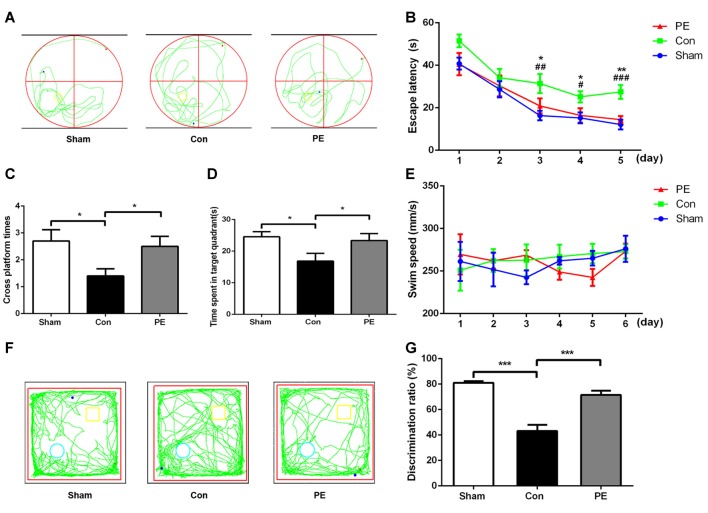
Physical exercise alleviated cognition impairment of rats with CCH. **(A)** Representative swimming path of each group. **(B)** Average escape latency of each group. **(C)** Times of crossing the platform of each group. **(D)** Time spent in the target quadrant of each group. **(E)** Average swimming speed of each group. **(F)** Representative moving tracks of each group in the novel object recognition (NOR) test. Yellow square: familiar objects, green circle: novel objects. **(G)** The novel object discrimination ratio of each group. Data represent the means ± SEM. *n* = 10. Sham vs. Con: ^#^*P* < 0.05, ^##^*P* < 0.01, ^###^*P* < 0.001; PE vs. Con: *P < 0.05, **P < 0.01, ***P < 0.001. Con, control; PE, physical exercise.

MWM and NOR tests were performed to evaluate the cognitive function of rats after 2VO. In the MWM test (Figures 2A–E), there was an impairment in learning abilities in the control group, as indicated by longer escape latency compared with the sham group on days 3–5. Nevertheless, the physical exercise group spent significantly less time to find the hidden platform compared with the control group on day 3 to day 5 (day 3: 20.89 ± 3.47 s vs. 31.42 ± 4.47 s, day 4: 16.41 ± 3.40 s vs. 25.17 ± 2.68 s, day 5: 14.42 ± 1.63 s vs. 27.48 ± 3.27 s, respectively; Figure 2B). Based on the spatial probe test, compared with the sham group, the times of crossing the platform and the dwell time in the target quadrant decreased in the control group. However, physical exercise reversed this deficit, as shown by the greater numbers of platform crossings (2.50 ± 0.37 vs. 1.40 ± 0.27; Figure 2C) and longer time spent in the target quadrant (23.36 ± 2.18 s vs. 16.83 ± 2.46 s; Figure 2D). There was no significant difference among groups in swimming speed during the experiments (Figure 2E, Supplementary Figure S1E), indicating that 2VO did not cause motor dysfunction in rats. The results showed that physical exercise improved the spatial learning and memory capacities of 2VO rats, but it had no significant effect on the rats received sham operation (Supplementary Figures S1A–D). In the NOR test (Figures 2F,G), the capacity to recognize novel objects was decreased in the control group compared with the sham group. However, the physical exercise group showed a significant reversal regarding a decrease in the novel object discrimination index relative to the control group (72 ± 3% vs. 43 ± 5%, Figure 2G), and it also had no significant effect on the rats received sham operation (Supplementary Figures S1F,G).These results suggest that physical exercise also improved the non-spatial memory of 2VO rats.

### Physical Exercise Alleviated Myelin Damage in Rats with Chronic Cerebral Hypoperfusion

**Figure 3:**
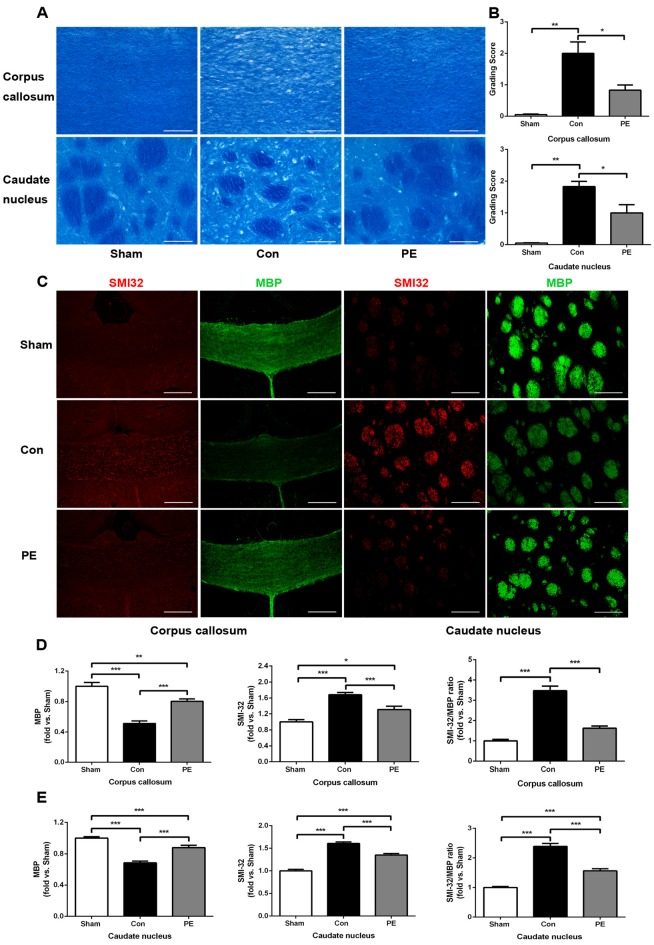
Physical exercise reduced myelin damage in rats with CCH. **(A)** Representative images of luxol fast blue (LFB) staining in the corpus callosum and caudate nucleus at 28 days after 2VO. Bar = 20 μm. **(B)** White matter (WM) grading-score histograms of each group. **(C)** Representative images of SMI32 (red) and MBP (green) immunofluorescence double labeling in corpus callosum (Bar = 50 μm) and caudate nucleus (Bar = 20 μm). **(D,E)** The fluorescence intensity of SMI32, MBP and SMI32/MBP of each group. Calculated as fold change over sham. Data represent the means ± SEM. *n* = 6. *P < 0.05, **P < 0.01, ***P < 0.001. MBP, myelin basic protein; SMI32, non-phosphorylated neurofilament H protein.

LFB staining was performed to observe the effect of physical exercise on myelin integrity. The results showed that myelin damage (including myelin fiber disorder, vacuolization, or myelin sheath loss) appeared in the corpus callosum and caudate nucleus of the control group at 28 days after 2VO, compared with the sham group (Figures 3A,B). In addition, relative to the control group, physical exercise significantly reduced myelin damage, as shown by decreasing WM grading scores (Figure 3B). Whereas it had no significant effect on the rats received sham operation as there does not exist really cerebral ischemia and myelin injury (Supplementary Figures S2A,B). Furthermore we evaluated the pathological changes of myelin by MBP and SMI32 immunofluorescence double labeling (Figures 3C–E). MBP is a marker of myelin integrity, whereas SMI32, an abnormally non-phosphorylated neurofilament protein, is a marker of axonal injury. The results showed that SMI32 was rarely expressed in the sham group, while the control group showed increased expression of SMI32 and a loss of MBP intensity. In contrast, physical exercise enhanced MBP immunostaining and decreased the fluorescence intensity ratio of SMI32/MBP (Figures 3D,E), indicating that physical exercise could reduce myelin loss and axonal damage in rats with CCH.

### Physical Exercise Enhanced the Proliferation and Differentiation of OPCs

**Figure 4:**
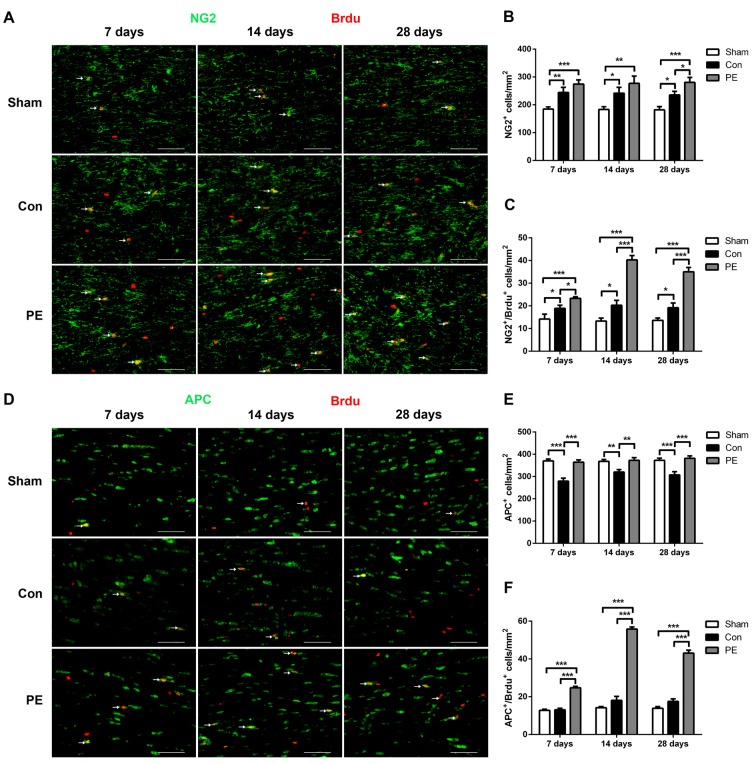
Physical exercise promoted oligodendrocyte progenitor cells (OPCs) proliferation and differentiation. **(A)** Representative images of NG2 (green) and 5-bromo-20-deoxyuridine (BrdU) (red) immunofluorescence double staining in the corpus callosum at 7, 14 and 28 days after 2VO. Bar = 20 μm. **(B,C)** Quantification of NG2^+^ and NG2^+^/BrdU^+^ cells. **(D)** Representative images of APC (green) and BrdU (red) immunofluorescence double staining in the corpus callosum at 7, 14 and 28 days after 2VO. Bar = 20 μm. **(E,F)** Quantification of APC^+^ and APC^+^/BrdU^+^ cells. Data represent the means ± SEM. *n* = 6. *P < 0.05, **P < 0.01, ***P < 0.001.

To explore the effect of physical exercise on proliferation and differentiation of OPCs in rats with CCH, BrdU intraperitoneal injection was conducted to label proliferating cells. Rats were sacrificed at different time points of 7, 14 and 28 days to conduct NG2/BrdU (Figures 4A–C) and APC/BrdU (Figures 4D–F) double-labeling immunofluorescence tests. The results showed that the NG2^+^ OPCs and NG2^+^/BrdU^+^ newborn OPCs in the control group were increased at all time points compared with the sham group. Physical exercise further augmented the expression of NG2^+^ cells at 28 days (280.56 ± 17.92/mm^2^ vs. 235.00 ± 13.64/mm^2^; Figure 4B), and NG2^+^/BrdU^+^ cells at all time points relative to the control group (7 days: 23.33 ± 0.70/mm^2^ vs. 18.89 ± 1.37/mm^2^, 14 days: 40.28 ± 1.92/mm^2^ vs. 20.28 ± 2.16/mm^2^, 28 days: 35.00 ± 1.94/mm^2^ vs. 19.17 ± 2.11/mm^2^, respectively; Figure 4C). Furthermore, relative to the sham group, the APC^+^ oligodendrocytes were significantly lower at each time point and there was no significant increase in APC^+^/BrdU^+^ positive cells at the same time in the control group. However, compared with the control group, physical exercise rescued the diminution of APC^+^ cells (7 days: 364.44 ± 10.53/mm^2^ vs. 279.17 ± 13.35/mm^2^, 14 days: 372.78 ± 11.99/mm^2^ vs. 320.28 ± 10.58/mm^2^, 28 days: 381.94 ± 10.59/mm^2^ vs. 307.22 ± 14.85/mm^2^, respectively; Figure 4E) and promoted OPC differentiation, as reflected by the higher number of APC^+^/BrdU^+^ newborn oligodendrocytes at each time point (7 days: 24.72 ± 0.75/mm^2^ vs. 13.06 ± 0.82/mm^2^, 14 days: 55.83 ± 1.09/mm^2^ vs. 18.06 ± 2.11/mm^2^, 28 days: 43.06 ± 1.57/mm^2^ vs. 17.50 ± 1.36/mm^2^, respectively; Figure 4F). Additionally, physical exercise seemly increased the number of NG2^+^/BrdU^+^ positive newborn OPCs and the APC^+^/BrdU^+^ positive newborn oligodendrocytes in the rats received sham operation, but did not have significant difference (Supplementary Figures S3A–F).

### Physical Exercise Promoted the Transformation of Microglia Phenotype from M1 to M2

**Figure 5:**
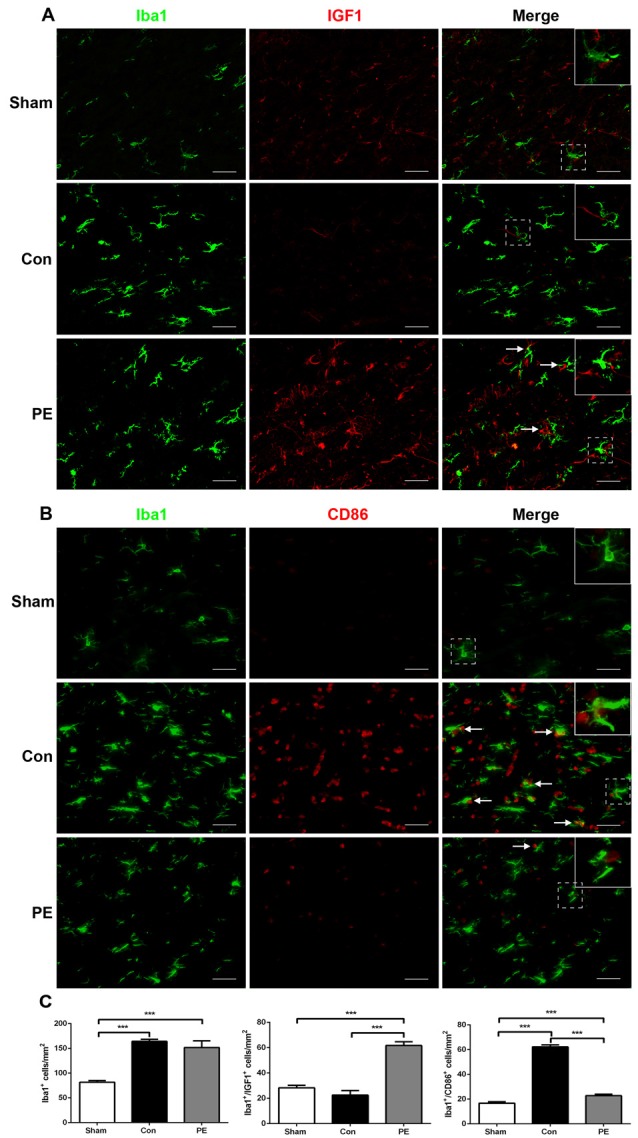
Physical exercise switched microglia from M1 to M2 phenotype. **(A)** Representative images of Iba1 (green) and IGF1 (red) immunofluorescence double staining in the corpus callosum at 28 days after 2VO. Bar = 20 μm. **(B)** Representative images of Iba1 (green) and CD86 (red) immunofluorescence double staining in the corpus callosum at 28 days after 2VO. Bar = 20 μm. **(C)** Quantification of Iba1^+^ microglia, IGF1^+^/Iba1^+^ M2 and CD86^+^/Iba1^+^ M1 microglia. Data represent the means ± SEM. *n* = 6. ***P < 0.001. IGF1, insulin-like growth factor 1.

**Figure 6:**
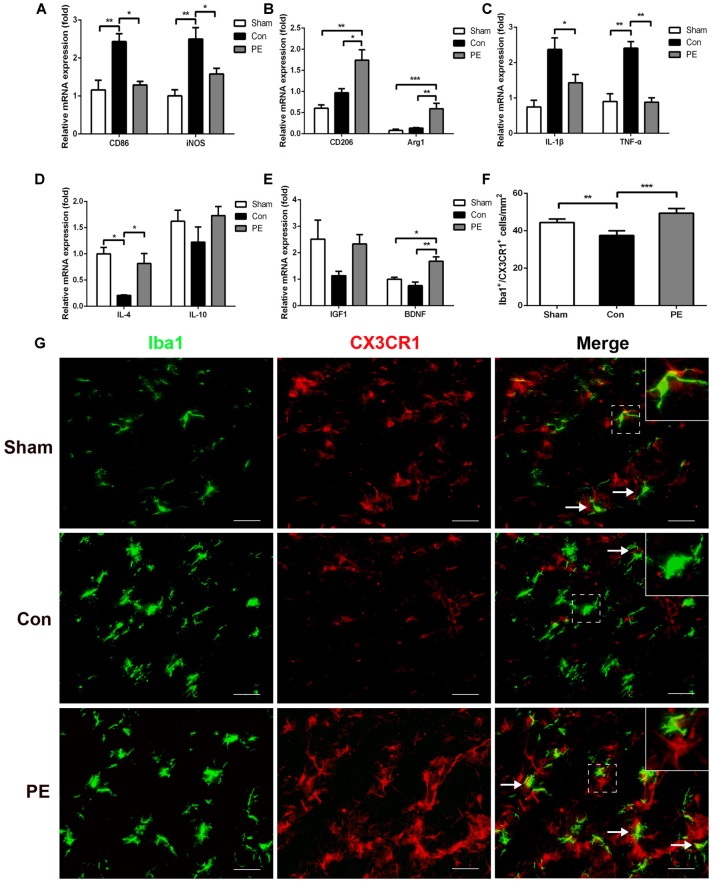
Physical exercise promoted neuroprotective response and CX3CR1 expression in microglia. The mRNA level of M1 (*CD86, iNOS*) **(A)** and M2 (*CD206, Arg1*) **(B)** microglia markers, inflammatory factors (*TNF-α, IL-1β, IL-4, IL-10*) **(C,D)** and neurotrophic factors (*BDNF, IGF1*) **(E)** in the corpus callosum were analyzed by real-time quantitative polymerase chain reaction of each group. *n* = 5. **(G)** Representative images of Iba1 (green) and CX3CR1 (red) immunofluorescence double staining in the corpus callosum at 28 days after 2VO. Bar = 20 μm. **(F)** Quantification of CX3CR1^+^/Iba1^+^ microglia. Data represent the means ± SEM. *n* = 6. *P < 0.05, **P < 0.01, ***P < 0.001. BDNF, brain-derived neurotrophic factor.

Microglia exhibits distinct morphological phenotypes, including ramified microglia with smaller cell bodies and longer processes, intermediate microglia with larger cell bodies and shorter processes, and ameboid microglia with large cell bodies and almost no processes evident (Kreutzberg, 1996; Thored et al., 2009; Xiong et al., 2016; Supplementary Figures S2C–E). In the sham group, most microglia were in a resting state and physical exercise had no significant effect on the phenotype change of the rats received sham operation (Supplementary Figures S2F,G). To further validate the effect of physical exercise on the phenotypic transformation of microglia in rats with CCH, IGF1/Iba1 and CD86/Iba1 immunofluorescence double-labeled staining were detected at 28 days after 2VO (Figures 5A–C). The results showed that more Iba1^+^ cells were enriched in control and physical exercise groups compared with the sham group. In addition, relative to the control group, physical exercise upregulated the number of IGF1/Iba1 positive M2 microglia (61.67 ± 2.94/mm^2^ vs. 22.50 ± 3.60/mm^2^) and decreased the number of CD86/Iba1 positive M1 microglia (22.78 ± 1.23/mm^2^ vs. 62.22 ± 1.68/mm^2^; Figure 5C). Furthermore, RT-PCR was used to evaluate the mRNA level of M1 microglia markers (*CD86, iNOS*; Figure 6A), M2 markers (*CD206, Arg1*; Figure 6B), inflammatory factors (*TNF-α, IL-1β, IL-4, IL-10*; Figures 6C,D) and neurotrophic factors (*IGF1, BDNF*; Figure 6E). In accordance with the above findings, the level of M1 markers (*CD86, iNOS*) and pro-inflammatory factors (*TNF-α, IL-1β*) were significantly increased in the control group compared with the sham group. M2 microglia markers (*CD206, Arg1*), anti-inflammatory factors (*IL-4, IL-10*) and neurotrophic factors *(IGF1, BDNF*) were downregulated at the same time. In contrast, physical exercise shifted microglia polarization from M1 toward M2 with the elevated level of M2 microglia markers (*CD206, Arg1*), anti-inflammatory factors (*IL-4, IL-10*), neurotrophic factors (*IGF1, BDNF*) and downregulated expression of M1 microglia markers (*CD86, iNOS*) and pro-inflammatory factors (*TNF-α, IL-1β*).

### Physical Exercise Improved CX3CL1/CX3CR1 Axis Expression and Regulated Phosphorylation Levels of JNK and ERK

**Figure 7:**
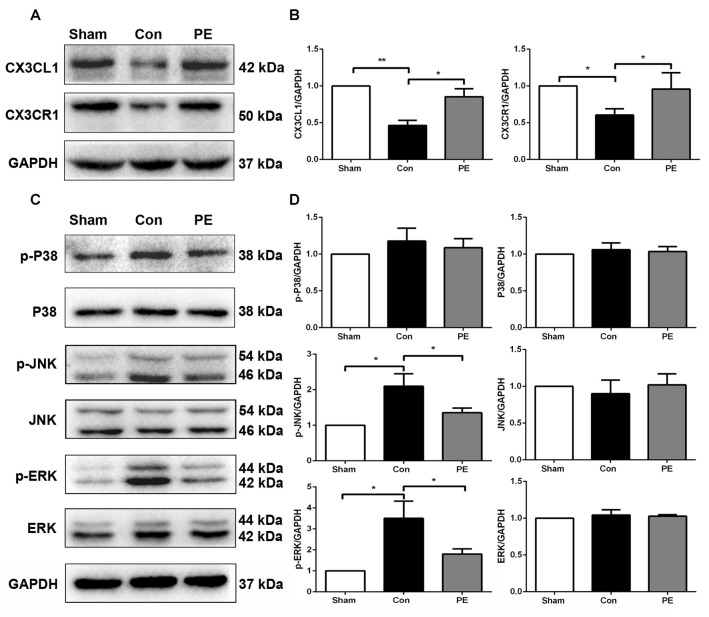
Physical exercise increased the expression of CX3CL1/CX3CR1 axis and decreased ERK and JNK phosphorylation. **(A)** Representative Western blot of CX3CL1 and CX3CR1 expression. **(B)** Densitometry analyses of CX3CL1 and CX3CR1 expression normalized to GAPDH. **(C)** Representative Western blotting of P-P38, P38, P-JNK, JNK, P-ERK1/2 and ERK1/2 expression. **(D)** Densitometry analyses of P-P38, P38, P-JNK, JNK, P-ERK1/2 and ERK1/2 expression normalized to GAPDH. Data represent the means ± SEM. *P < 0.05, **P < 0.01. *n* = 5.

To explore the effects of physical exercise on the CX3CL1/CX3CR1 axis and its downstream MAPKs signaling in rats with CCH, CX3CR1/Iba1 immunofluorescence double-labeled staining was performed to observe the expression of the CX3CR1 specifically in microglia at 28 days after 2VO (Figure 6G). The results showed that the CX3CR1^+^/Iba1^+^ positive microglia decreased in the control group compared with the sham group, whereas physical exercise significantly increased the expression of CX3CR1^+^/Iba1^+^ positive microglia (Figure 6F). Furthermore, western blot analysis was performed to detect protein expression of CX3CL1, CX3CR1, P-P38, P38, P-JNK, JNK, P-ERK1/2 and ERK1/2 in the corpus callosum of rats at 28 days after 2VO. Based on our results, the control group showed downregulated the expression of CX3CL1 and CX3CR1 and increased phosphorylation of ERK1/2 and JNK compared with the sham group. However, physical exercise could ameliorate these negative effects by significantly augmenting the expression of CX3CL1 and CX3CR1 (Figures 7A,B), and decreasing the phosphorylation level of ERK and JNK (Figures 7C,D).

## Discussion

Oligodendrocyte is vulnerable to ischemic injury, and decrease in CBF can lead to oligodendrocytes loss and myelin damage (Dewar et al., 2003; Tomimoto et al., 2003). In this study, we found that the CBF of local cortical regions decreased to about 30% of the baseline level after 2VO in rats. The results of LFB staining and SMI32/MBP immunofluorescence double labeling confirmed myelin damage. However, physical exercise improved cognitive function in rats with CCH and reduced myelin loss and axonal damage by decreasing WM damage scores, as well as downregulating the fluorescence intensity ratio of SMI32 to MBP. Similar results have been found in clinical studies suggesting that physical exercise improved WM integrity of patients with cerebral small vessel disease (Gons et al., 2013). Additionally, progressive resistance physical training could reverse WM hyperintensities progression and improve overall cognitive function in patients with dementia prodrome mild cognitive impairment (Suo et al., 2016). Therefore, the improvement of cognition observed with physical exercise may be associated with an enhancement of myelin integrity.

In response to demyelination, OPCs may generate new myelinating oligodendrocytes through proliferation and differentiation to replace myelin loss, which is an essential mechanism for myelin repair (Gensert and Goldman, 1997; Franklin and Ffrench-Constant, 2008; Miyamoto et al., 2010). Our findings supported that rats maintained a significantly higher number of NG2^+^/BrdU^+^ newborn OPCs after 2VO, indicating that myelin damage caused by ischemic injury could promote OPCs proliferation. Previous studies have suggested that OPCs are increased in the brains of patients with vascular dementia and rats with CCH (Miyamoto et al., 2010). However, the number of APC^+^/BrdU^+^ newborn oligodendrocytes after 2VO in our study did not increase, which may be attributed to the ischemic-induced toxic microenvironment that blocked OPCs differentiation or caused newborn OPCs death. It is known that the process of OPCs differentiation is most vulnerable, which may explain the failure of myelin repair during demyelination (Kuhlmann et al., 2008; Miyamoto et al., 2013; Back and Rosenberg, 2014). We confirmed that physical exercise further enhanced OPCs proliferation and significantly increased the expression of APC^+^/BrdU^+^ newborn oligodendrocytes. Physical exercise is known to have a positive effect on OPCs proliferation, and OPCs may be most affected by exercise in the medial prefrontal cortex (Mandyam et al., 2007; Kuhlmann et al., 2008). An important finding showed that complex wheel exercise triggered OPCs proliferation and oligodendrocytes generation, and these changes in turn are crucial for optimal motor learning (McKenzie et al., 2014). Therefore, physical exercise enhanced myelin integrity in rats with CCH, possibly through promoting OPCs proliferation and differentiation.

Additionally, the mechanism of the effects of physical exercise on oligodendrocytogenesis under cerebral ischemia and physiological conditions may be different, as the proliferation and differentiation of OPCs were affected by the pathological microenvironment induced by cerebral ischemia.

It has been shown that microglia can exert destructive or protective effects on oligodendrocytes depending on the M1 or M2 polarization status (Praet et al., 2014; Wang et al., 2015). M2 microglia can drive OPCs differentiation and prevent newborn oligodendrocytes apoptosis, which is an essential mechanism for effective remyelination. Depletion or aging-related reduction of M2 microglia would cause remyelination failure (Miron et al., 2013). Previous studies showed that rosiglitazone can shift microglia polarization from M1 to M2 and promote oligodendrocytegenesis and remyelination, thereby repairing WM integrity and improving cognitive function of mice after MCAO (Han et al., 2015). In this study, we found that physical exercise decreased the expression of CD86/Iba1 positive M1 microglia, upregulated the expression of Iba1/IGF1 positive M2 microglia and promoted a protective response in rats after 2VO. Furthermore, RT-PCR indicated that physical exercise hindered 2VO-induced M1 microglia activation by attenuating the gene expression of M1 markers (*CD86, iNOS*) and pro-inflammatory factors (*TNF-α, IL-1β*). In addition, the level of M2 markers (*CD206, Arg1*), anti-inflammatory factor (*IL-4, IL-10*) and neurotrophic factors (*IGF1, BDNF*) in the physical exercise group were upregulated. In agreement with our results, previous study found that wheel running mitigated microglia proliferation and increased the number of Iba1/IGF1 positive M2 microglia in the hippocampal dentate gyrus of aged mice. This was association with running-induced neurogenesis (Kohman et al., 2012). Therefore, physical exercise promoted oligodendrocytegenesis and remyelination may be associated with its regulation of microglia polarization from M1 toward M2.

The CX3CL1/CX3CR1 axis plays an important role in regulating microglia activation and controlling their neurotoxicity. However, its protective or deleterious effects following ischemic injury remain controversial. Previous studies reported that CX3CL1 deficiency could create a protective microenvironment and reduce infarct lesions of mice after MCAO (Fumagalli et al., 2013). However, other studies have shown that the expression of CX3CL1 was reduced in the hippocampus of aged rats, leading to microglia chronic activation and neurogenesis suppression (Bachstetter et al., 2011). Additionally, the CX3CL1/CX3CR1 axis may switch microglia to a neuroprotective type with enhanced phagocytic activity and promote myelin debris clearance and remyelination in multiple sclerosis (Lampron et al., 2015). In our study, we demonstrated that the expression of CX3CL1 and CX3CR1 in the corpus callosum was reduced, whereas physical exercise enhanced the expression of CX3CL1/CX3CR1 axis in rats with CCH. Furthermore, physical exercise significantly reduced ERK and JNK phosphorylation. The MAPK family, particularly ERK1/2, JNK and P38 signals are CX3CL1/CX3CR-modulated inflammatory pathways and play an important role in inflammation regulation (Réaux-Le Goazigo et al., 2013). Currently, the exact causes of the controversial results of the CX3CL1/CX3CR1 remain unclear and may be partly due to differences in the experimental conditions, animal models and measurement time (Liu et al., 2015). In addition, the effects of CX3CL1/CX3CR1 axis depend on the context and microglia response (Ferreira et al., 2014). Physical exercise may create a favorable microenvironment in which the CX3CL1/CX3CR1 axis could promote microglia to a positive response by mediating ERK- and JNK-dependent pathways.

The study has several limitations. Our future study will focus on the detailed understanding of the direct involvement of microglia and OPCs in physical exercise linked to improvement of cognitive function, and the time window of exercise on cognitive improvement and its underlying mechanism(s) in CCH.

In conclusion, our study increases our understanding of the mechanism of physical exercise improving cognitive function in rats with CCH, possibly be related to microglia phenotype modulation and then enhancement of oligodendrocytegenesis and remyelination. Moreover, the CX3CL1/CX3CR1 axis may play an important role in this process by mediating ERK- and JNK-dependent pathways.

## Author Contributions

XH, TJ, LZ and HZ conceived and designed experiments. TJ, LZ and XP performed experiments and data acquisition. XC, LL and JL analyzed and interpreted data. XH, TJ, LZ and HZ wrote the article. All authors read the final manuscript and approved it for publication.

## Conflict of Interest Statement

The authors declare that the research was conducted in the absence of any commercial or financial relationships that could be construed as a potential conflict of interest.

## Supplementary Material

TABLE S1 The mortality rates of the sham, sham+PE group, 2VO control group and 2VO+PE group.

FIGURE S1 Physical exercise has no significant effect on the cognitive function of the rats received sham operation. **(A)** Representative swimming path of the sham and sham+PE group. **(B)** Average escape latency of the sham and sham+PE group. **(C)** Times of crossing the platform of the sham and sham+PE group. **(D)** Time spent in the target quadrant of the sham and sham+PE group. **(E)** Average swimming speed of the sham and sham+PE group. **(F)** Representative moving tracks of the sham and sham+PE group in the novel object recognition (NOR) test. Yellow square: familiar objects, green circle: novel objects. **(G)** The novel object discrimination ratio of the sham and sham+PE group. Data represent the means ± SEM. *n* = 10.

FIGURE S2 Physical exercise has no significant effect on the myelin integrity and microglia morphological phenotype of the rats received sham operation. **(A)** Representative images of luxol fast blue (LFB) staining of the sham and sham+PE group in the corpus callosum and caudate nucleus at 28 days after 2VO. Bar = 20 μm. **(B)** White matter (WM) grading-score histograms of the sham and sham+PE group. **(C–E)** Microglia exhibits distinct morphological phenotypes, including ramified microglia **(C)**, intermediate microglia **(D)** and ameboid microglia **(E)**. Bar = 10 μm. **(F)** Representative images of Iba1 immunohistochemistry staining of the sham and sham+PE group in the corpus callosum at 28 days after 2VO. Bar = 20 μm. **(G)** Quantification of three different phenotypes microglia of the sham and sham+PE group. Data represent the means ± standard error of mean (SEM). *n* = 6.

FIGURE S3 Physical exercise has no significant effect on oligodendrocyte progenitor cells (OPCs) proliferation and differentiation of the rats received sham operation. **(A)** Representative images of NG2 (green) and 5-bromo-20-deoxyuridine (BrdU; red) immunofluorescence double staining in the corpus callosum of the sham and sham+PE group at 7, 14 and 28 days after 2VO. Bar = 20 μm. **(B,C)** Quantification of NG2^+^ and NG2^+^/BrdU^+^ cells. **(D)** Representative images of APC (green) and BrdU (red) immunofluorescence double staining in the corpus callosum of the sham and sham+PE group at 7, 14 and 28 days after 2VO. Bar = 20 μm. **(E,F)** Quantification of APC^+^ and APC^+^/BrdU^+^ cells. Data represent the means ± standard error of mean (SEM). *n* = 6.
