# Supplementary material for: Physical Exercise Improves Cognitive Function Together with Microglia Phenotype Modulation and Remyelination in Chronic Cerebral Hypoperfusion
Source: Front Cell Neurosci. 2017 Dec 22;11:404. doi: 10.3389/fncel.2017.00404 (PMC5743796; doi:10.3389/fncel.2017.00404)
Supplement: TABLE S1 — The mortality rates of the sham, sham+PE group, 2VO control group and 2VO+PE group. [file Table_1.docx]

**Supplementary table 1**

**The mortality rates of the sham, sham+PE group, 2VO control group and 2VO+PE group.**

| Groups | Operated rats | Dead rats | Mortality |
| --- | --- | --- | --- |
| Sham | 28 | 1 | 3.57% |
| Sham+PE | 22 | 0 | 0% |
| 2VO control | 28 | 5 | 17.86% |
| 2VO+PE | 28 | 6 | 21.43% |

Sham VS sham+PE (P > 0.05); 2VO control VS 2VO+PE (P > 0.05).
