# Supplementary figures and images for: Physical Exercise Improves Cognitive Function Together with Microglia Phenotype Modulation and Remyelination in Chronic Cerebral Hypoperfusion
Source: Front Cell Neurosci. 2017 Dec 22;11:404. doi: 10.3389/fncel.2017.00404 (PMC5743796; doi:10.3389/fncel.2017.00404)

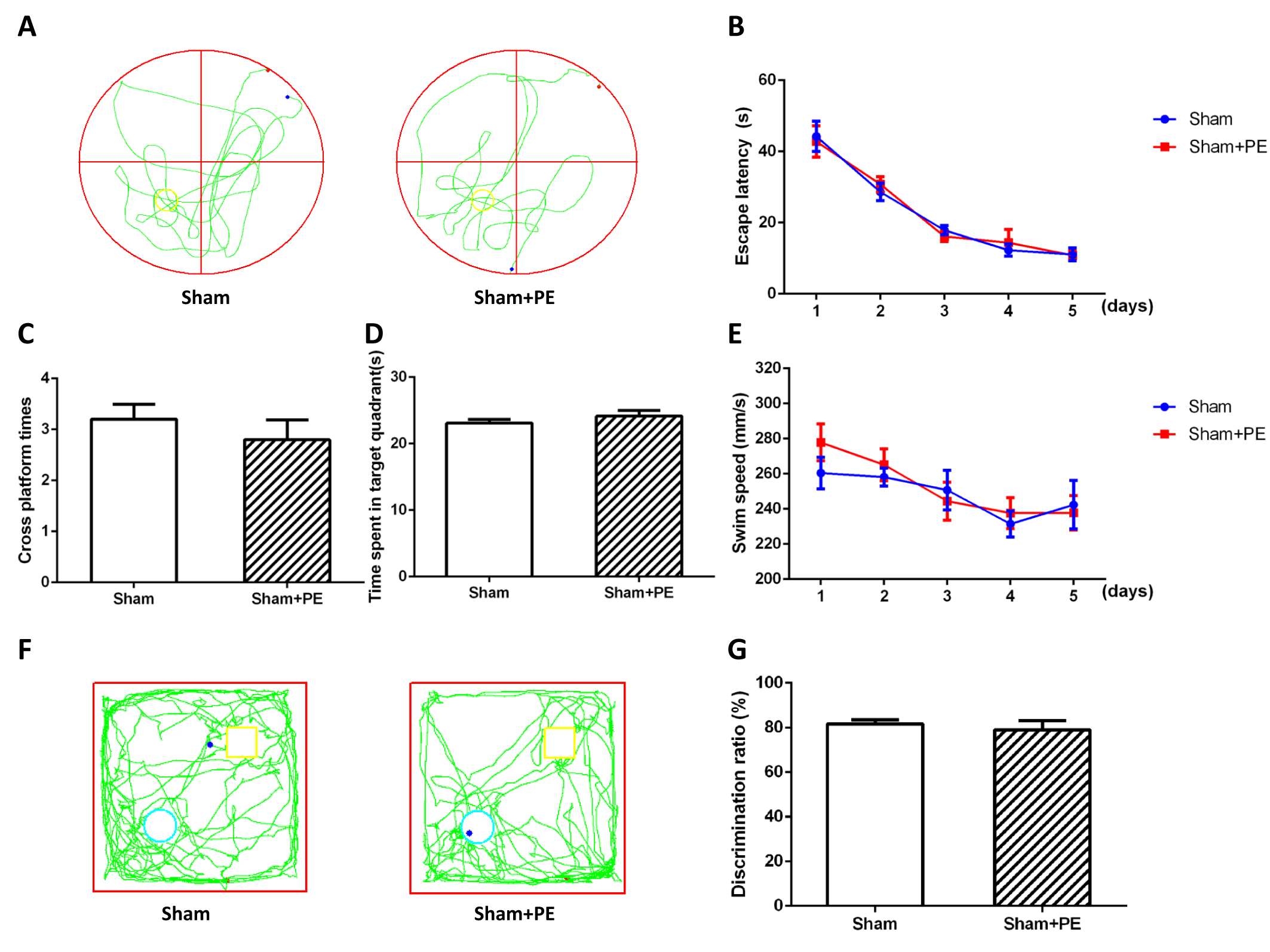

Supplement: FIGURE S1 — Physical exercise has no significant effect on the cognitive function of the rats received sham operation. (A) Representative swimming path of the sham and sham+PE group. (B) Average escape latency of the sham and sham+PE group. (C) Times of crossing the platform of the sham and sham+PE group. (D) Time spent in the target quadrant of the sham and sham+PE group. (E) Average swimming speed of the sham and sham+PE group. (F) Representative moving tracks of the sham and sham+PE group in the novel object recognition (NOR) test. Yellow square: familiar objects, green circle: novel objects. (G) The novel object discrimination ratio of the sham and sham+PE group. Data represent the means ± SEM. n = 10. [file Image_1.tif]

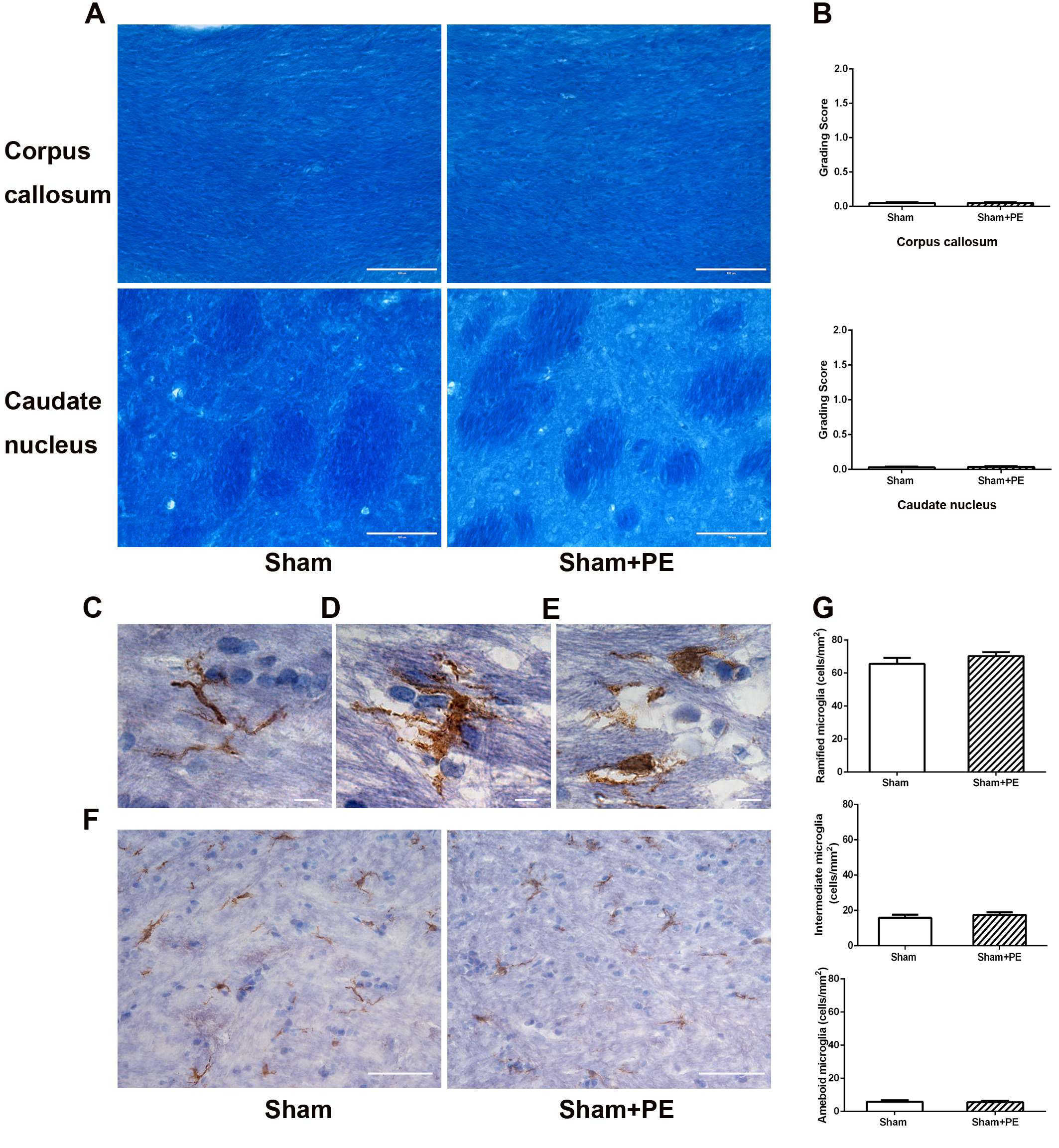

Supplement: FIGURE S2 — Physical exercise has no significant effect on the myelin integrity and microglia morphological phenotype of the rats received sham operation. (A) Representative images of luxol fast blue (LFB) staining of the sham and sham+PE group in the corpus callosum and caudate nucleus at 28 days after 2VO. Bar = 20 μm. (B) White matter (WM) grading-score histograms of the sham and sham+PE group. (C–E) Microglia exhibits distinct morphological phenotypes, including ramified microglia (C), intermediate microglia (D) and ameboid microglia (E). Bar = 10 μm. (F) Representative images of Iba1 immunohistochemistry staining of the sham and sham+PE group in the corpus callosum at 28 days after 2VO. Bar = 20 μm. (G) Quantification of three different phenotypes microglia of the sham and sham+PE group. Data represent the means ± standard error of mean (SEM). n = 6. [file Image_2.tif]

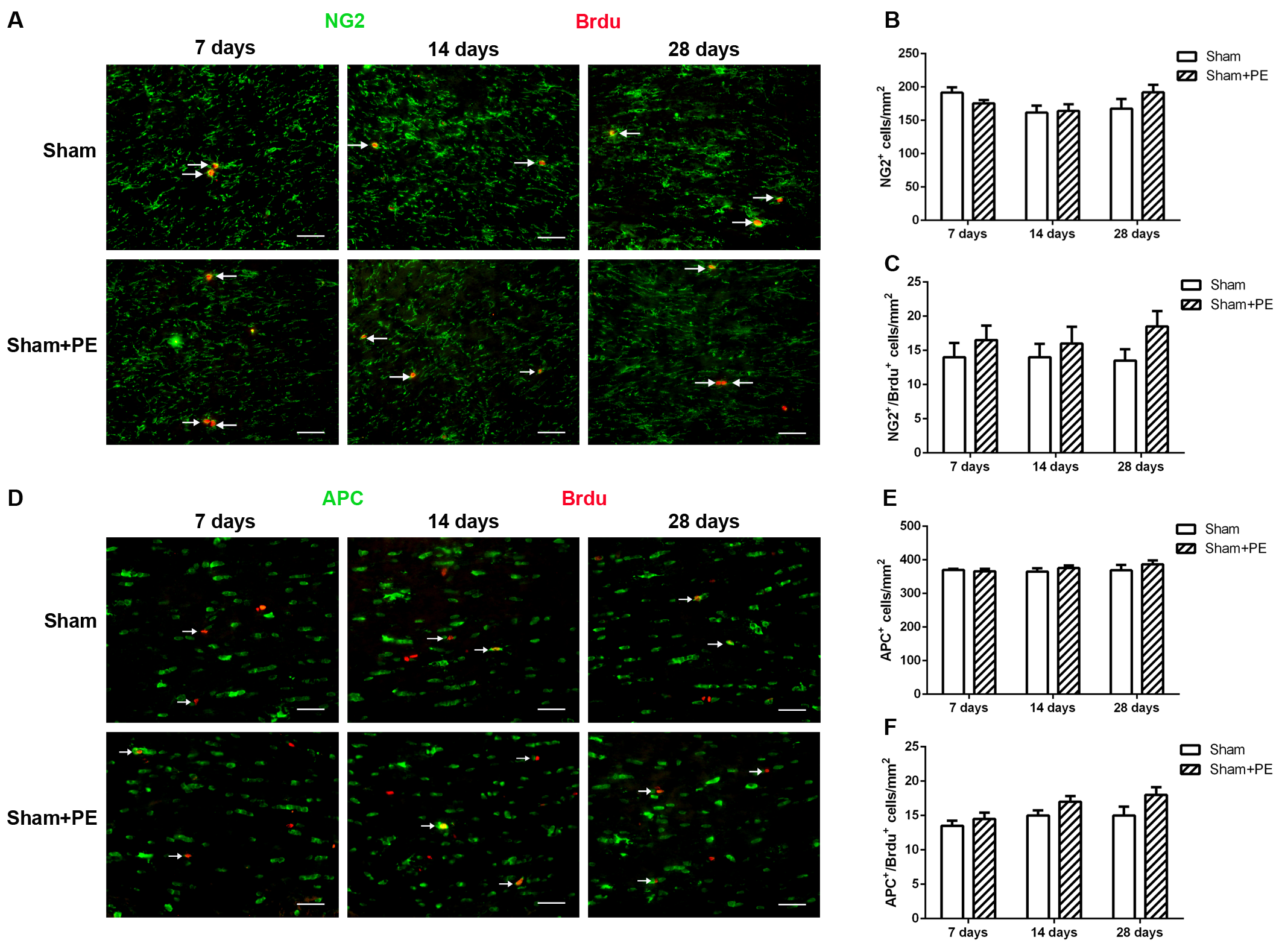

Supplement: FIGURE S3 — Physical exercise has no significant effect on oligodendrocyte progenitor cells (OPCs) proliferation and differentiation of the rats received sham operation. (A) Representative images of NG2 (green) and 5-bromo-20-deoxyuridine (BrdU; red) immunofluorescence double staining in the corpus callosum of the sham and sham+PE group at 7, 14 and 28 days after 2VO. Bar = 20 μm. (B,C) Quantification of NG2+ and NG2+/BrdU+ cells. (D) Representative images of APC (green) and BrdU (red) immunofluorescence double staining in the corpus callosum of the sham and sham+PE group at 7, 14 and 28 days after 2VO. Bar = 20 μm. (E,F) Quantification of APC+ and APC+/BrdU+ cells. Data represent the means ± standard error of mean (SEM). n = 6. [file Image_3.tif]
